# LncRNA CAR10 Upregulates PDPK1 to Promote Cervical Cancer Development by Sponging miR-125b-5p

**DOI:** 10.1155/2020/4351671

**Published:** 2020-01-03

**Authors:** Tingting Hu, Qian Zhang, Lingxue Gao

**Affiliations:** ^1^Department of Gynecology, Zibo Maternal and Child Health Hospital, Zibo 255000, Shandong, China; ^2^Department of Gynecology and Obstetrics, Tongzhou Maternal and Child Health Hospital of Beijing, Beijing 101101, China; ^3^Department of Gynecology, Qingdao Women and Children's Hospital, Qingdao 266034, Shandong, China

## Abstract

Cervical cancer is one of the malignant tumors that seriously threaten women's health. The mechanism of development needs to be deeply studied. In recent years, lncRNA has been identified as one of the important factors affecting the malignant progression of tumors. In this study, we illustrated the important mechanism of lncRNA CAR10 in the development of cervical cancer. We found that CAR10 is significantly increased in4 cervical cancer tissues and cells, which can promote the proliferation of cervical cancer cells in vitro and in vivo, indicating that CAR10 is involved in the progression of cervical cancer as an oncogene. Further studies showed that CAR10 is a target gene of miR-125b-5p, and miR-125b-5p can inhibit the effect of CAR10 on the proliferation of cervical cancer cells. In addition, we also found that 3-phosphoinositide-dependent protein kinase 1 (PDPK1) is also a target gene of miR-125b-5p, and CAR10 can upregulate the expression level of PDPK1. The results showed that CAR10 acts as a ceRNA to upregulate the expression of PDPK1 by sponging miR-125b-5p. Knockdown of PDPK1 can inhibit the effect of CAR10 on cervical cancer cells. Our study demonstrates that, based on ceRNA mechanism, CAR10/miR-125b-5p/PDPK1 network can regulate the proliferation of cervical cancer cells and play an important role in the development of cervical cancer. In addition, our study also suggests that intervention of CAR10/miR-125b-5p/PDPK1 network may be a new strategy for targeted therapy of cervical cancer.

## 1. Introduction

The incidence and mortality of cervical cancer are the highest in female reproductive system tumors [1]. Especially in developing countries, the age of cervical cancer occurrence is younger, which is a serious threat to women's health and life. About 500,000 new cases of cervical cancer are diagnosed each year, accounting for 5% of all new cancer cases, of which about 85% occur in developing countries where medical resources are relatively scarce [2]. The persistent infection of human papillomavirus (HPV) is an important cause of cervical cancer [3]. Although the application of HPV vaccine can prevent cervical cancer, the treatment and prognosis of cervical cancer are still urgently needed to be studied. Therefore, it is of great significance to study the pathogenesis and biological mechanism of cervical cancer.

Long noncoding RNAs (lncRNAs) are a class of RNA molecules that are more than 200 nucleotides in length and do not have the function of encoding proteins [4]. The structure and sequence of lncRNA are somewhat conserved, and recent studies have found that it is not as originally thought to have no biological function. More and more studies have shown that lncRNA can participate in many physiological processes and various diseases [5–7]. In recent years, more and more lncRNAs have been found to be abnormally expressed and play an important role in various tumor tissues. For example, the expression of lncRNA CCAT1 in various digestive system tumors is abnormal. CCAT1 was significantly upregulated in colorectal cancer, which was closely related to the prognosis of patients [8]. In addition, the expression of CCTA1 is also regulated by the oncogene c-Myc to promote the proliferation of cancer cells [9]. The expression of lncRNA ZFAS1 is increased in colorectal cancer tissues. Knockdown of ZFAS1 can block the cell cycle of colorectal cancer cells in the G1 phase, thereby inhibiting the proliferation of colon cancer cells [10, 11]. LncRNA TINCR is downregulated in colorectal cancer, and overexpression of TINCR can inhibit the metastasis and proliferation of cancer cells [12]. LncRNA DQ786243 has been shown to regulate the proliferation and apoptosis of colon cancer cells in vitro and in vivo [13]. The expression of lncRNA MALAT1 was significantly elevated in various malignant tumors such as lung cancer, renal cancer, and liver cancer, which was closely related to the poor prognosis of patients [14]. With the development of high-throughput sequencing technology, more and more lncRNAs have been discovered, but the mechanism of most lncRNAs in tumors remains unclear and needs to be studied in depth. LncRNA CAR10 is a new tumor-associated lncRNA discovered in recent years. The current study found that CAR10 is upregulated in lung cancer tissues and participates in the development of lung cancer as an oncogene [15, 16]. However, the relevance of CAR10 to other tumors is unclear. Based on the Lnc2Cancer 2.0 database, we found that CAR10 was one of the significantly differential expression genes in cervical cancer. After detection by RT-qPCR of cervical cancer tissues, we confirmed that CAR10 was upregulated in cervical cancer. In this study, we revealed the mechanism of CAR10 in the development of cervical cancer.

Our study reveals that CAR10 is involved in the development of cervical cancer as an oncogene. CAR10 is upregulated in cervical cancer tissues and promotes the proliferation of cervical cancer cells in vitro and in vivo. Further studies reveal that CAR10 can promote the expression of PDPK1 by sponging miR-125b-5p, which may be one of the mechanisms that promote the proliferation of cervical cancer cells. Our research confirms that CAR10 involves in the development of cervical cancer as an oncogene and provides a new strategy for targeted therapy of cervical cancer.

## 2. Materials and Methods

### 2.1. Plasmids, miRNAs, and siRNAs

The overexpression plasmids pcDNA-CAR10 and lentiviral overexpression plasmid Lenti-CAR10 were constructed by GenScript Company (Nanjing, China). Luciferase reporter plasmids include wild-type and mutant pGLO-CAR10, as well as wild-type and mutant pGLO-PDPK1 3′UTR, both constructed by GenScript Company (Nanjing, China). The mimic and inhibitor of miR-125b-5p and PDPK1 siRNA were obtained from Genepharma Company (Shanghai, China).

### 2.2. Cervical Tissues

Cervical cancer tissues and paracancerous tissues were collected from Qingdao Women and Children's Hospital. Patient specimens were obtained with informed consent, and the present study was approved by the Ethics Board of Qingdao Women and Children's Hospital and was based on all relevant principles of the Declaration of Helsinki.

### 2.3. Cell Culture and Transfection

The cells used in this study included cervical epithelial cells HUCEC and cervical cancer cell line Caski, C33A, HeLa, and SiHa. All the cells were obtained from the Cell Bank of Type Culture Collection of the Chinese Academy of Sciences (Shanghai, China). The cells were cultured in DMEM (HyClone), containing 10% FBS (fetal bovine serum) (Gibco), penicillin (100 U/ml), and streptomycin (100 mg/ml) at 37°C, 5% CO_2_. The protocol of plasmids and miRNA transfection into cells using Lipofectamine 2000 reagent (Invitrogen) are referred to in the manufacturer's instruction.

### 2.4. Reverse Transcription and Quantitative Polymerase Chain Reaction (RT-qPCR)

Tissues and cells were lysed by TRIzol reagent (Invitrogen), according to the manufacturer's instructions. Total RNA was extracted and reverse-transcribed into cDNA using QuantiTect Rev. Transcription Kit (QIAGEN). The generated cDNA was used as template to detect the expression of genes using QuantiNova SYBR Green PCR Kit (QIAGEN). The RT primer for miR-125b-5p was GTCGTATCCAGTGCAGGGTCCGAGGTATTCGCACTGGATACGACCTCACAA; U6: GTCGTATCCAGTGCAGGGTCCGAGGTATTCGCACTGGATACGACAAAT-ATG; miR-1249-5p: GTCGTATCCAGTGCAGGGTCCGAGGTATTCGCACTGGATAACTTG; miR-2277-5p: GTCGTATCCAGTGCAGGGTCCGAGGTATTCGCACTGGA-TGACTGG; miR-3192-5p: GTCGTATCCAGTGCAGGGTCCGAGGTATTCGCACTGGATTTCCAC; miR-3663-5p: GTCGTATCCAGTGCAGGGTCCGAGGTATTCGCACT-GGATCCGAGC; miR-3692-5p: GTCGTATCCAGTGCAGGGTCCGAGGTATTCGCACTGGATCAGTAT.

The primers for qPCR included miR-125b-5p (5′-TCCCTGAGACCCTAAC-3′ and 5′-GTGCAGGGTCCGAGGT-3′), miR-1249-5p (5′-AGGAGGGAGGAGAUGGGC-3′ and 5′-GTGCAGGGTCCGAGGT-3′), miR-2277-5p (5′-AGCGCGGGCTGAGCGCTG-3′ and 5′-GTGCAGGGTCCGAGGT-3′), miR-3192-5p (5′-TCTGGGAGGTTGTAGC-A-3′ and 5′-GTGCAGGGTCCGAGGT-3′), miR-3663-5p (5′-GCTGGTCTGCGTGGT-3′ and 5′-GTGCAGGGTCCGAGGT-3′), miR-3692-5p (5′-CCTGCTGGTCAGGAGTGG-3′ and 5′-GTGCAGGGTCCGAGGT-3′), U6 (5′-CTCGCTTCGGCAGCACA-3′ and 5′-AACGCTTCACGAATT-TGCGT-3′), CAR10 (5′-TCAGTGCTGCTCCTGAGAGA-3′ and 5′-CAGCCAGAGACCAGTCATCA-3′), cyclin A1 (5′-ACATGGATGAACTAGAGCAGGG-3′ and 5′-GAGTGTGCCGGTGTCTACTT-3′), cyclin B1 (5′-AATAAGGCGAAGATCAACATGGC-3′ and 5′-TTTGTTACCAATG-TCCCCAAGAG-3′), CDK2 (5′-CCAGGAGTTACTTCTATGCCTGA-3′ and 5′-TTCATCCAGGGGAGGTACAAC-3′), cyclin A2 (5′-CGCTGGCGGTACTGAAGTC-3′ and 5′-GAGGAACGGTGACATGCTCAT-3′), and GAPDH (5′-CTGGGCTACACTGAGCACC-3′ and 5′-AAGTGGTCG-TTGAGGGCAATG-3′).

### 2.5. Western Blotting

Cells or tissues were lysed using RIPA buffer. BCA Protein Assay Kit II (Biovision, USA) was used to measure protein concentration. The antibodies include cyclin A1 (Proteintech, USA), cyclin B1 (Proteintech, USA), CDK2 (Proteintech, USA), PDPK1 (Proteintech, USA), and *β*-actin (Proteintech, USA). HRP-labeled goat anti-rabbit and goat anti-mouse secondary antibodies were purchased from Cell Signaling Technology. The process of western blotting was referred to in the previous article [17].

### 2.6. RNA-Binding Protein Immunoprecipitation (RIP) Assay

The Ago2-RIP assay was performed using RNA Immunoprecipitation Kit (Millipore, Germany) according to the manufacturer's protocol. The magnetic beads were incubated with AGO2 antibody or IgG. The immunoprecipitated RNAs were further analyzed by qRT-PCR. Total RNAs served as input control.

### 2.7. Measurement of Cell Proliferation

The cell proliferation was detected by colony formation assay and CCK-8 assay. For the CCK-8 assay, cells of each group were seeded in 96-well cell culture plate, and the proliferation ability of cells was assessed by CCK-8 kit (Dojindo, Japan) by a microplate reader (BD Company, USA), according to the manufacturer's protocol. For the colony formation assay, in brief, cells of each group were seeded in a 6-well cell culture plate, and after about one week, cells were stained and counted by crystal violet using a microscope.

### 2.8. Flow Cytometry Analysis

Cell apoptosis was detected by flow cytometry using Annexin V-FITC/PI Apoptosis Detection Kit (BD, USA). According to the manufacturer's protocol, cells were labeled with Annexin V-FITC/PI and analyzed by flow cytometry.

#### 2.8.1. Measurement of Cell Migration

Wound healing and transwell assays were performed to measure the ability of cell migration. For the wound healing assay, cells were seeded in 6-well plates and cultured for 48 hours. A pipette tip (200 *μ*l) was used to make a straight scratch. Cell wound images were taken by a microscope at 0 and 48 hours for examining wound healing. For the transwell migration assay, 2 × 10^4^ cells were seeded in the upper transwell chamber insert (Corning, USA). The lower chamber was filled with a complete culture medium containing 10% serum. Cells were allowed to migrate towards the serum gradient for 24 hours. Migrated cells were stained with 1% crystal violet and counted using a phase-contrast microscope. Five random fields were counted per experiment.

### 2.9. Luciferase Assay

Cells of each group were transfected with report gene plasmids using Lipofectamine 2000. After transfection for 48 hours, the relative luciferase value was detected by GloMax 20/20 Luminometer (Promega, USA) using Dual-Luciferase reporter Assay Kit (Promega, USA).

### 2.10. Nude Mouse Xenograft Model

Stably overexpressing cells were constructed in C33A cells using lentiviral overexpression plasmids Lenti-CAR10 and Lenti-NC by GenScript Company (Nanjing, China). Nude mice were randomly divided into two groups. Each group included 10 nude mice. A number of 1 × 10^7^ stably overexpressing C33A cells were injected into the back of nude mice and the growth of neoplasm in nude mice was observed and recorded. When the neoplasm grows to a suitable size, the nude mice are sacrificed by cervical dislocation, and the neoplasm is collected, and the weight of the neoplasm is measured. The neoplasm is stored in liquid nitrogen to use to detect the expression level of the target protein. The in vivo study was approved by the Ethics Board of Qingdao Women and Children's Hospital.

### 2.11. Statistical Analysis

Each experiment was repeated three times. The data were expressed as the mean ± standard deviation (SD). The statistical analysis between groups was performed using one-way ANOVA method. *p* < 0.05 was considered statistically significant.

## 3. Results

### 3.1. CAR10 Is Upregulated in Cervical Cancer Tissues and Cells and Negatively Correlated with Disease-Free Survival of Patients with Cervical Cancer

**Figure 1:**
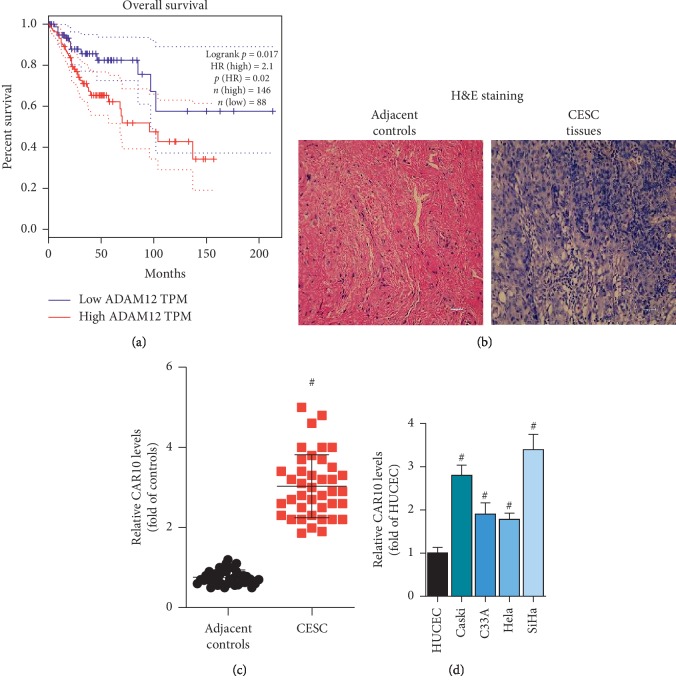
CAR10 is upregulated in cervical cancer tissues and cells. (a) Bioinformatics analysis showed a negative correlation between CAR10 and disease-free survival of patients with cervical cancer. We collected 40 cervical cancer tissues and paracancerous tissues (b) and detected mRNA expression levels of CAR10 in cervical cancer tissues and adjacent tissues by RT-qPCR (c), ^#^*p* < 0.05, compared with adjacent controls. (d) In normal cervical epithelial cells HUCEC, as well as cervical cancer cells Caski, C33A, HeLa, and SiHa, the expression level of CAR10 was detected by RT-qPCR, ^#^*p* < 0.05, compared with HUCEC.

There is no research showing the mechanism of CAR10 in the development of cervical cancer. We found through bioinformatics analysis that CAR10 was negatively correlated with disease-free survival in patients with cervical cancer (Figure 1(a)). Then, we collected 40 cases of cervical cancer tissue and its adjacent tissues (Figure 1(b)). The results of RT-qPCR showed that the expression level of CAR10 was significantly upregulated in cervical cancer tissues, which was closely related to the differentiation, grade, tumor size, and lymph node metastasis (Figure 1(c), Table 1). In addition, compared with normal cervical epithelial cells HUCEC, the expression level of CAR10 in cervical cancer cells Caski, C33A, HeLa, and SiHa was also significantly increased (Figure 1(d)). These results suggest that CAR10 is upregulated during the development of cervical cancer and may be associated with the prognosis of cervical cancer.

### 3.2. CAR10 Can Promote the Proliferation of Cervical Cancer Cells In Vitro and In Vivo

**Figure 2:**
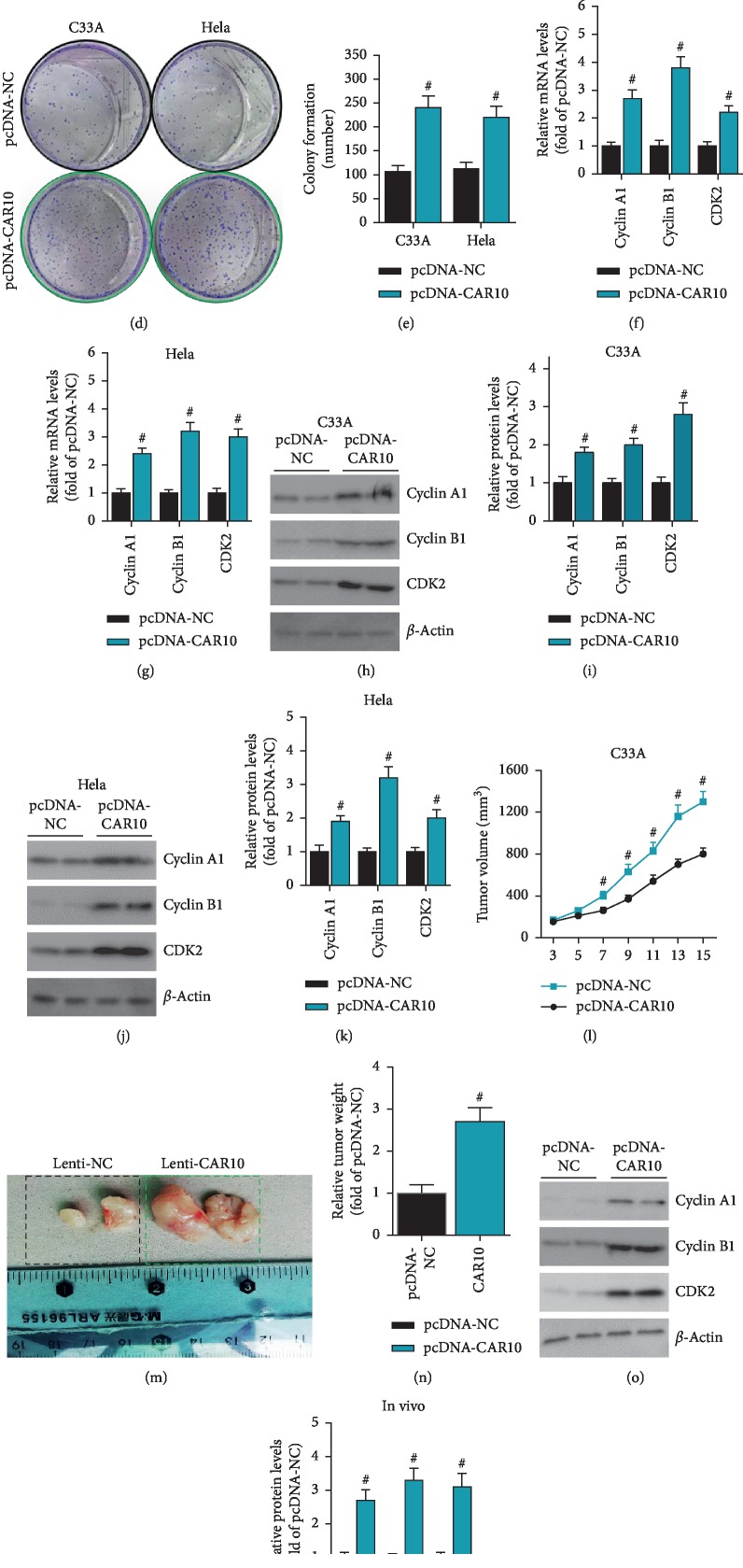
CAR10 promotes the proliferation of cervical cancer cells in vitro and in vivo. We transfected the overexpression plasmid pcDNA-CAR10 and the negative control pcDNA-NC into C33A and HeLa cells and detected the expression level of CAR10 by RT-qPCR (a), ^#^*p* < 0.05, compared with pcDNA-NC. Proliferative capacity of cervical cancer cells C33A and HeLa cells was examined by CCK-8 assay (b, c) and colony formation experiments (d, e), ^#^*p* < 0.05, compared with pcDNA-NC. The mRNA expression levels of cyclin A1, cyclin B1, and CDK2 were detected by RT-qPCR (f, g), ^#^*p* < 0.05, compared with pcDNA-NC. The protein expression levels of cyclin A1, cyclin B1, and CDK2 were detected by western blot (h, i), ^#^*p* < 0.05, compared with pcDNA-NC. (l) The CAR10 stably overexpressing C33A cells were injected subcutaneously into nude mice, and the growth of the neoplasms was monitored in real time, ^#^*p* < 0.05, compared with pcDNA-NC. (m, n) After the nude mice were sacrificed, the neoplasms were removed and the weight was counted. ^#^*p* < 0.05, compared with pcDNA-NC. (o, p) Western blot was used to detect the protein expression levels of cyclin A1, cyclin B1, and CDK2 in the neoplasms, ^#^*p* < 0.05, compared with pcDNA-NC.

We have observed that CAR10 is upregulated in cervical cancer tissues and cells. To observe the effect of upregulated CAR10 on the biological behavior of cervical cancer cells, we constructed the overexpression plasmid pcDNA-CAR10, and pcDNA-NC was used as a negative control vector. After C33A and HeLa cells were transfected with pcDNA-CAR10 and pcDNA-NC (Figure 2(a)), the proliferation ability of the cells was observed by CCK-8 assay and colony formation assays. The results of CCK-8 assay showed that overexpression of CAR10 significantly enhanced the proliferation of C33A and HeLa cells (Figures 2(b) and 2(c)). The results of the colony formation assay were consistent with the CCK-8 assay (Figures 2(d) and 2(e)). Both cyclin A1 and cyclin B1 are members of the cyclin family. They promote and coordinate the cell cycle by combining with cyclin-dependent kinases (CDKs) [18]. Therefore, upregulation of cyclin A1, cyclin B1, and CDK2 expression levels can promote cell proliferation. In C33A and HeLa cells, RT-qPCR results showed that overexpression of CAR10 significantly upregulated the mRNA expression levels of cyclin A1, cyclin B1, and CDK2 (Figures 2(f) and 2(g)). Western blot results also showed that overexpression of CAR10 significantly upregulated the protein expression levels of cyclin A1, cyclin B1, and CDK2 (Figures 2(h)–2(k)). This is consistent with the results of the CCK-8 assay and the clone formation assay. Furthermore, we observed the effect of CAR10 on cervical cancer cells by in vivo study. We constructed a cervical cancer cell line C33A stably overexpressing CAR10 and then inoculated subcutaneously into nude mice. After a certain period of observation, we found that overexpression of CAR10 can significantly promote the growth rate and weight of cervical cancer neoplasms (Figures 2(l)–2(n)). Moreover, the protein expression of cyclin A1, cyclin B1, and CDK2 in cervical cancer neoplasms of CAR10 overexpression group was also significantly increased (Figures 2(o) and 2(p)). These results indicate that upregulated CAR10 acts as an oncogene to promote the progression of cervical cancer.

### 3.3. CAR10 Promotes Cell Migration and Inhibits Cell Apoptosis in Cervical Cancer

**Figure 3:**
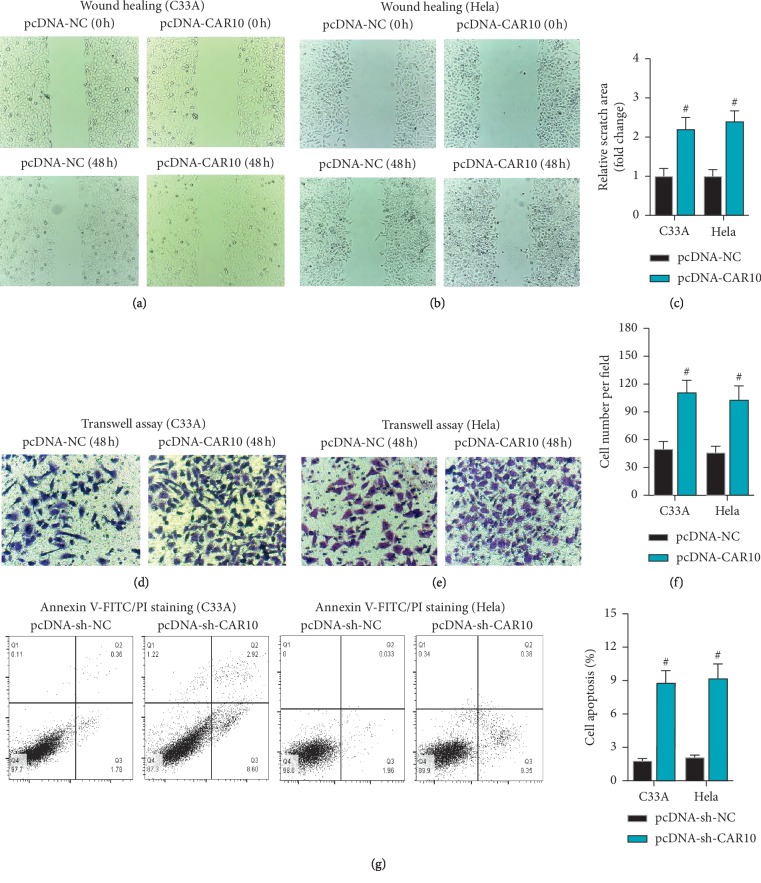
CAR10 promotes cell migration and inhibits cell apoptosis in cervical cancer. The effect of CAR10 overexpression on the migration of C33A (a) and HeLa (b, c) cells was detected by wound healing assay. The effect of CAR10 overexpression on the migration of C33A (d) and HeLa (e, f) cells was detected by transwell assay. (g) The effect of CAR10 overexpression on the apoptosis of C33A and HeLa cells was detected by flow cytometry assay. ^#^*p* < 0.05, compared with pcDNA-NC.

The effect of CAR10 on the migration and apoptosis of cervical cancer cells was unknown. In this study, wound healing and transwell assay were used to detect the ability of cell migration. The results of wound healing assay showed that CAR10 overexpression enhanced the ability of migration in C33A and HeLa cells (Figures 3(a)–3(c)). In addition, the results of transwell assay were consistent with wound healing assay (Figures 3(d)–3(f)). Furthermore, we also detected the effect of CAR10 on the cell apoptosis in cervical cancer by flow cytometry. The results showed that CAR10 overexpression inhibited cell apoptosis in cervical cancer (Figure 3(g)). These results further proved that CAR10 acted as an oncogene in cervical cancer.

### 3.4. CAR10 Is the Target Gene of miR-125b-5p

**Figure 4:**
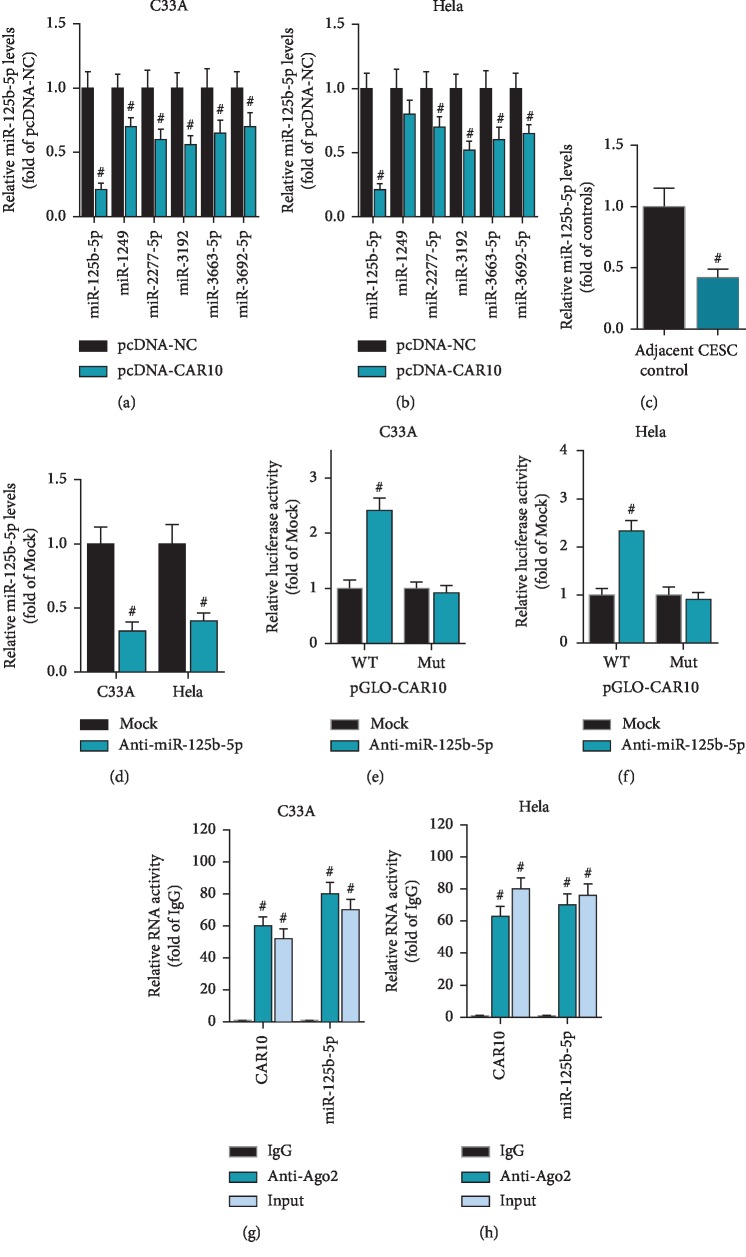
CAR10 is the target gene of miR-125b-5p. (a, b) The effect of CAR10 overexpression on miR-125b-5p, miR-1249, miR-2277-5p, miR-3192, miR-3663-5p, and miR-3692-5p expression level was detected by RT-qPCR in C33A and HeLa cells, ^#^*p* < 0.05, compared with pcDNA-NC. (c) Detection of miR-125b-5p expression levels in cervical cancer tissues and paracancerous tissues collected by RT-qPCR, ^#^*p* < 0.05, compared with adjacent controls. (d) The miR-125b-5p inhibitor (anti-miR-125b-5p) and the negative control Mock were transfected into C33A and HeLa, and the expression level of miR-125b-5p was detected by RT-qPCR, ^#^*p* < 0.05, compared with Mock. (e, f) The effect of anti-miR-125b-5p on the activity of pGLO-CAR10 WT and pGLO-CAR10 Mut was examined by luciferase assay, ^#^*p* < 0.05, compared with Mock. (g, h) The interaction of miR-125b-5p and CAR10 was detected by Ago2-RIP-qPCR assay, ^#^*p* < 0.05, compared with IgG.

Through bioinformatics prediction, we found that CAR10 contains the binding sequence of miR-125b-5p. In C33A and HeLa cells, overexpression of CAR10 significantly inhibited the expression levels of miR-125b-5p, miR-1249, miR-2277-5p, miR-3192, miR-3663-5p, and miR-3692-5p, in which the inhibitory effect on miR-125b-5p was most significant (Figures 4(a) and 4(b)). RT-qPCR results showed that the expression level of miR-125b-5p was significantly decreased in cervical cancer tissues (Figure 4(c)). To test whether CAR10 is a target gene of miR-125b-5p, we synthesized an inhibitor of miR-125b-5p (anti-miR-125b-5p) (Figure 4(d)). Based on the predicted binding sites, we designed reporter plasmid of CAR10 containing the wild-type binding sequence (WT) or the mutant binding sequence (Mut). The luciferase assay showed that anti-miR-125b-5p can significantly enhance the activity of wild-type CAR10 reporter gene (pGLO-CAR10 WT), while it has no effect on the mutant CAR10 reporter gene (pGLO-CAR10 Mut) in C33A and HeLa cells (Figures 4(e) and 4(f)). The miRNA forms an RNA-inducing silencing complex (RISC) with the Dicer enzyme and Ago 2 protein, thereby binding and inhibiting the expression of the target gene. Therefore, if CAR10 is a target gene of miR-125b-5p, miR-125b-5p binds to CAR10 with Dicer and Ago 2. miR-125b-5p and CAR10 were simultaneously detected by RIP-qPCR assay using Ago 2 antibody. As we predicted, the results of the RIP-qPCR experiment showed that miR-125b-5p may interact with CAR10 (Figures 4(g) and 4(h)). These findings indicate that CAR10 is a target gene of miR-125b-5p.

### 3.5. CAR10 Acts as a ceRNA to Upregulate the Expression of PDPK1 by Sponging miR-125b-5p

**Figure 5:**
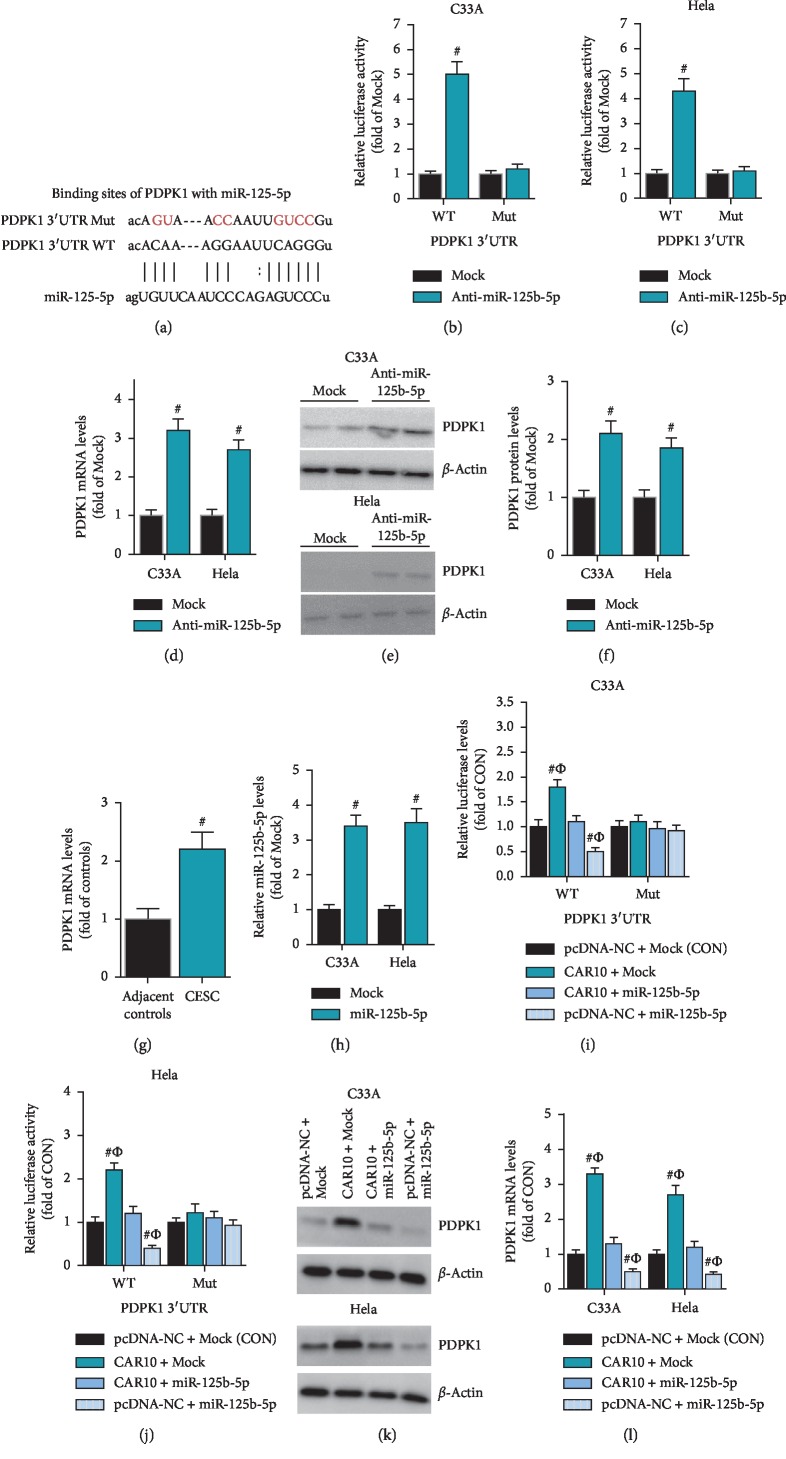
CAR10 acted as a ceRNA and upregulated the expression of PDPK1 by sponging miR-125b-5p. (a) The binding site of miR-125b-5p to the 3′UTR of PDPK1 was predicted by bioinformatics, and the binding site was mutated to construct luciferase reporter gene plasmids. (b, c) The effect of anti-miR-125b-5p on the activity of PDPK1 3′UTR WT and PDPK1 3′ UTR Mut was examined by luciferase assay in C33A and HeLa cells, ^#^*p* < 0.05, compared with Mock. (d, e, f) The effects of anti-miR-125b-5p on the expression of PDPK1 mRNA and protein were detected by RT-qPCR and western blot, ^#^*p* < 0.05, compared with Mock. (g) The mRNA expression level of PDPK1 was detected by RT-qPCR in the collected cervical cancer tissues and adjacent tissues, ^#^*p* < 0.05, compared with adjacent controls. The miR-125b-5p mimic (miR-125b-5p) and the negative control Mock were synthesized and transfected into C33A and HeLa cells, and (h) the expression level of miR-125b-5p was detected by RT-qPCR, ^#^*p* < 0.05, compared with Mock. (i, j) The effect of miR-125b-5p and/or CAR10 overexpression on the activity of PDPK1 3′UTR WT and PDPK1 3′ UTR Mut was examined by luciferase assay, ^#^*p* < 0.05, compared with CON. ^Ф^*p* < 0.05, compared with CAR10 + miR-125b-5p. (k) The effect of miR-125b-5p and/or CAR10 overexpression on PDPK1 expression was observed by western blot and RT-qPCR, ^#^*p* < 0.05, compared with CON. ^Ф^*p* < 0.05, compared with CAR10 + miR-125b-5p.

By bioinformatics prediction, we found that the 3′UTR region of PDPK1 contains the binding sequence of miR-125b-5p. Based on the binding sequences, we constructed PDPK1 3′UTR reporter plasmids containing the wild-type binding sequence (PDPK1 3′UTR WT) and the mutant binding sequence (PDPK1 3′UTR Mut) (Figure 5(a)). In C33A and HeLa cells, anti-miR-125b-5p significantly increased the activity of PDPK1 3′UTR WT, but had no effect on the activity of PDPK1 3′UTR Mut (Figures 5(b) and 5(c)). Anti-miR-125b-5p also significantly upregulated the mRNA and protein expression levels of PDPK1 in C33A and HeLa cells (Figures 5(d)–5(f)). These results indicate that PDPK1 is a target gene of miR-125b-5p. RT-qPCR results showed that the expression level of PDPK1 in cervical cancer tissues was significantly reduced (Figure 5(g)). In this study, we have confirmed that CAR10 and PDPK1 are both target genes of miR-125b-5p. In order to observe whether CAR10 can act as a ceRNA to regulate the expression of PDPK1, we synthesized miR-125b-5p mimic and then transfected it into C33A and HeLa cells (Figure 5(h)). In C33A and HeLa cells, luciferase assay showed that miR-125b-5p can significantly inhibit the activity of PDPK1 3′UTR WT, while CAR10 can significantly enhance the activity of PDPK1 3′UTR WT and reverse the inhibitory effect of miR-125b-5p on PDPK1 3′UTR WT. However, they both have no effect on PDPK1 3′UTR Mut (Figures 5(i) and 5(j)). In C33A and HeLa cells, western blot results showed that miR-125b-5p can significantly inhibit the expression of PDPK1, while CAR10 can significantly increase the expression level of PDPK1 and significantly reverse the inhibitory effect of miR-125b-5p on PDPK1 (Figures 5(k) and 5(l)). These results indicate that CAR10 acts as a ceRNA to upregulate PDPK1 expression by sponging miR-125b-5p in cervical cancer cells.

### 3.6. Based on ceRNA Mechanism, the CAR10/miR-125b-5p/PDPK1 Network Regulates the Proliferation of Cervical Cancer Cells

**Figure 6:**
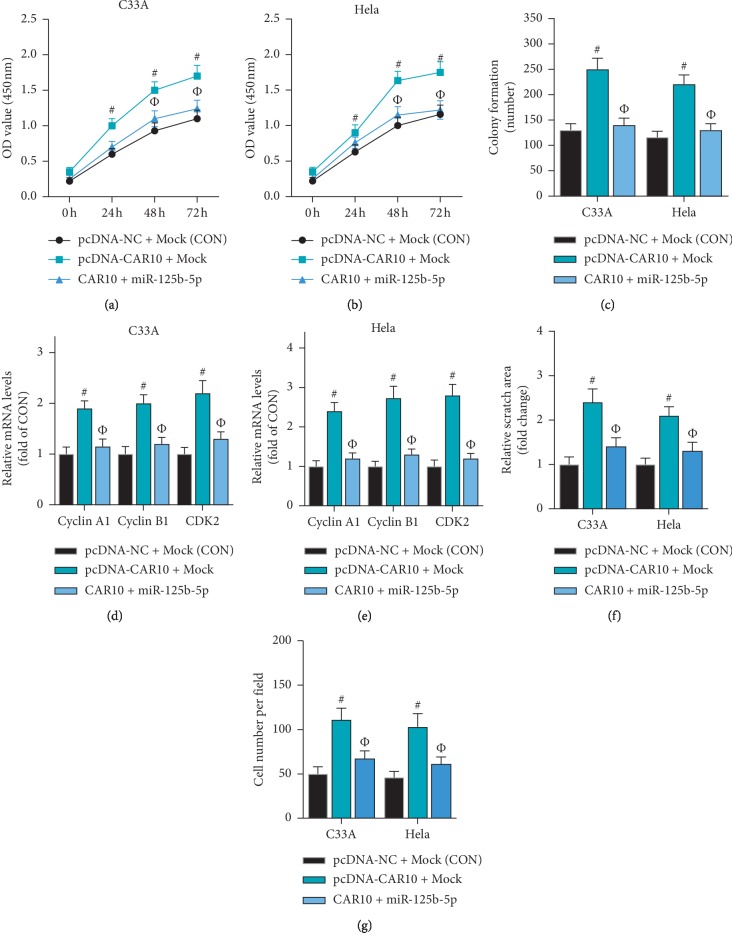
miR-125b-5p can reverse the effect of CAR10 on the proliferation of cervical cancer cells. The effect of CAR10 overexpression combined with miR-125b-5p on the proliferation of C33A and HeLa cells was examined by CCK-8 assay (a, b) and colony formation assay (c), ^#^*p* < 0.05, compared with CON. ^Ф^*p* < 0.05, compared with pcDNA-CAR10 + Mock. (d, e) The effects of CAR10 overexpression combined with miR-125b-5p on the mRNA expression levels of cyclin A1, cyclin B1, and CDK2 were detected by RT-qPCR in C33A and HeLa cells, ^#^*p* < 0.05, compared with CON. ^Ф^*p* < 0.05, compared with pcDNA-CAR10 + Mock. The effect of CAR10 overexpression combined with miR-125b-5p on the migration of C33A and HeLa cells was examined by wound healing assay (f) and transwell assay (g), ^#^*p* < 0.05, compared with CON. ^Ф^*p* < 0.05, compared with pcDNA-CAR10 + Mock.

In the present study, we have determined that CAR10 acts as a ceRNA to upregulate PDPK1 expression by sponging miR-125b-5p. Can the CAR10/miR-125b-5p/PDPK1 network regulate the proliferation of cervical cancer cells? In C33A and HeLa cells, overexpression of CAR10 significantly promoted cell proliferation, while miR-125b-5p reversed the effect of CAR10 overexpression on cell proliferation (Figures 6(a) and 6(b)). The results of the colony formation assay were consistent with the CCK-8 assay (Figure 6(c)). In addition, western blot results showed that miR-125b-5p can reverse the effect of CAR10 overexpression on the expression of cyclin A1, cyclin B1, and CDK2 (Figures 6(d) and 6(e)). Furthermore, the wound healing assay showed that miR-125b-5p can reverse the effect of CAR10 overexpression on promoting cell migration in C33A and HeLa cells (Figure 6(f)). The results of transwell assay were consistent with the wound healing assay (Figure 6(g)). These results indicate that CAR10 regulates cervical cancer cell proliferation and migration via miR-125b-5p.

**Figure 7:**
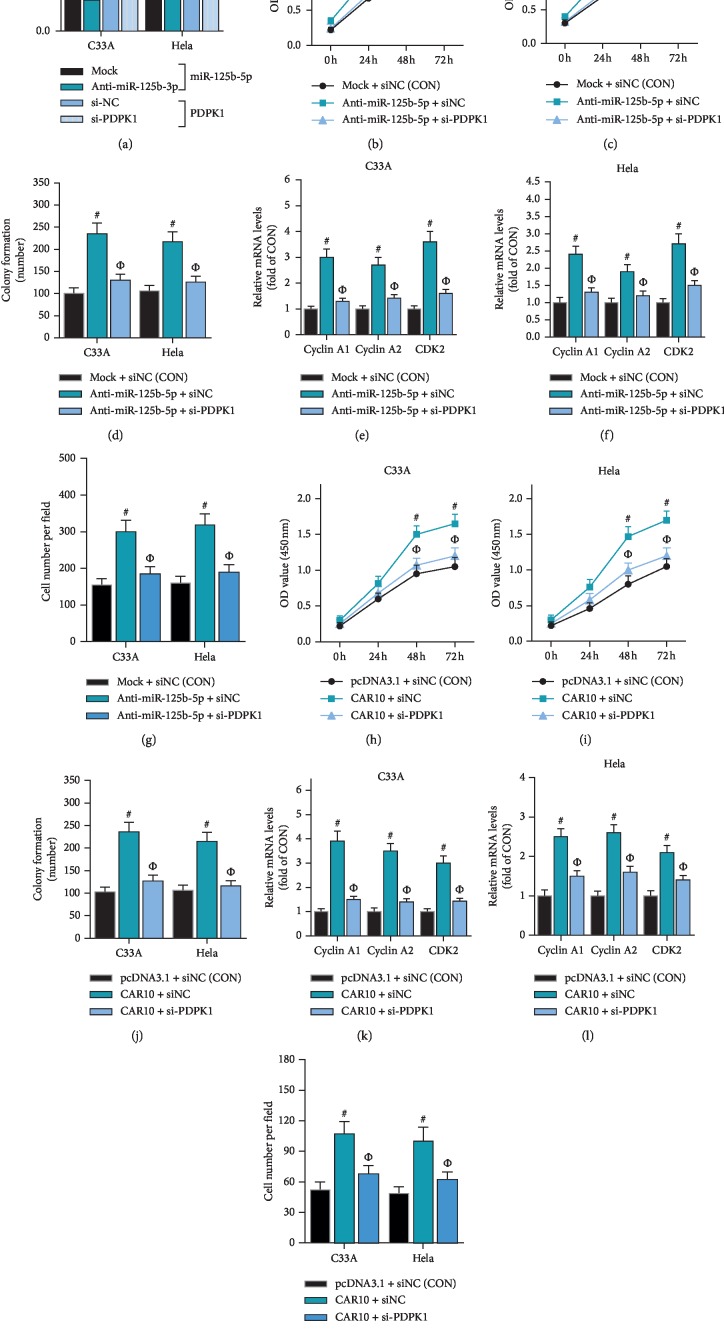
CAR10/miR-125b-5p/PDPK1 network regulates the proliferation of cervical cancer cells. miR-125b-5p inhibitors (anti-miR-125b-5p) or PDPK1 siRNA (si-PDPK1) was transfected into C33A and HeLa cells, respectively, (a) the expression level of miR-125b-5p and PDPK1 was detected by RT-qPCR, ^#^*p* < 0.05, compared with Mock. ^Ф^*p* < 0.05, compared with si-NC. (b, c, d) The effects of anti-miR-125b-5p and/or si-PDPK1 on cell proliferation were examined by CCK-8 assay and colony formation assay, ^#^*p* < 0.05, compared with CON. ^Ф^*p* < 0.05, compared with anti-miR-125b-5p + si-NC. (e, f) The effects of anti-miR-125b-5p and/or si-PDPK1 on the expression of cyclin A1, cyclin A2, and CDK2 were detected by RT-qPCR, ^#^*p* < 0.05, compared with CON. ^Ф^*p* < 0.05, compared with anti-miR-125b-5p + si-NC. (g) The effects of anti-miR-125b-5p and/or si-PDPK1 on cell migration were examined by transwell assay, ^#^*p* < 0.05, compared with CON. ^Ф^*p* < 0.05, compared with anti-miR-125b-5p + si-NC. (h, i, j) The effects of CAR10 overexpression and/or si-PDPK1 on cell proliferation were examined by CCK-8 assay and colony formation assay, ^#^*p* < 0.05, compared with CON. ^Ф^*p* < 0.05, compared with CAR10 + si-NC. (k, l) The effects of CAR10 overexpression and/or si-PDPK1 on the expression of cyclin A1, cyclin A2, and CDK2 were detected by RT-qPCR, ^#^*p* < 0.05, compared with CON. ^Ф^*p* < 0.05, compared with CAR10 + si-NC. (m) The effects of CAR10 overexpression and/or si-PDPK1 on cell migration were examined by transwell assay, ^#^*p* < 0.05, compared with CON. ^Ф^*p* < 0.05, compared with CAR10 + si-NC.

In C33A and HeLa cells, the effect of PDPK1 siRNA (si-PDPK1) combined with miR-125b-5p inhibitor (anti-miR-125b-5p) on cell proliferation was tested by CCK-8 assay and colony formation assay. The results of CCK-8 assay and colony formation assay showed that anti-miR-125b-5p can significantly enhance the proliferation of C33A and HeLa cells, while si-PDPK1 can reverse the effect of anti-miR-125b-5p on proliferation of cervical cancer cells (Figures 7(a)–7(d)). The results of RT-qPCR showed si-PDPK1 could reverse the effect of anti-miR-125b-5p on promoting the expression of cyclin A1, cyclin B1, and CDK2 (Figures 7(e) and 7(f)). Furthermore, the results of transwell assay showed that anti-miR-125b-5p can significantly enhance the migration of C33A and HeLa cells, while si-PDPK1 can reverse the effect of anti-miR-125b-5p on migration of cervical cancer cells (Figure 7(g)). These results indicate that the regulation of miR-125b-5p on proliferation of cervical cancer cells is associated with PDPK1. In addition, in C33A and HeLa cells, si-PDPK1 can reverse the effect of CAR10 overexpression on promoting the proliferation of cervical cancer cells (Figures 7(h)–7(j)) and upregulating the expression of cyclin A1, cyclin B1, and CDK2 (Figures 7(k) and 7(l)). Furthermore, the results of transwell assay showed that si-PDPK1 can reverse the effect of CAR10 overexpression on promoting the cell migration in C33A and HeLa cells (Figure 7(m)). These results indicate that the ceRNA mechanism-based CAR10/miR-125b-5p/PDPK1 network can regulate the proliferation of cervical cancer cells.

## 4. Discussion

Recent studies have shown that lncRNA regulates gene expression at epigenetic level, transcriptional level, and posttranscriptional level. There is increasing evidence that lncRNA plays a very important role in epigenetic regulation [19]. LncRNA mediates expression silencing of related genes by recruiting chromatin recombination complexes to specific sites. For example, lncRNA KCNQ1OT1 recruits histone methyltransferase G9a and PRC2 to regulate gene expression [20]. In the inflammatory response involved in the NF-KB signaling pathway, lncRNA TNFAIP3 binds to HMGB1 and forms NF-KB/HMGB1/lncRNA TNFAIP3 complex, which regulates HMGB1-related histone modifications and affects the expression of inflammatory factors [21]. The regulation of lncRNA at transcriptional and posttranscriptional levels is mainly through the following mechanisms. As a molecular sponge, lncRNA inhibits the inhibition of target genes by binding and sponging miRNAs. For example, lncRNA SOX21-AS1 blocks the inhibition of the target gene VDAC1 by miR-7 via sponging miR-7 [22]. LncRNA is capable of interacting with transcription factors, preventing transcription factors from binding to target gene promoters, and preventing expression of target genes. For example, lncRNA GAS5 can directly bind to glucocorticoid receptor, preventing it from binding to the corresponding DNA response elements, thereby inhibiting transcription [23, 24]. LncRNA can bind to RNA-binding protein and recruit it to the promoter of target gene, thereby regulating gene expression. For example, lncRNA FEZF1-AS1 can deplete K3K4me2 in the promoter region of p21 gene by recruiting LSD1, thereby inhibiting p2l expression [25]. LncRNA can also interact with splicing factors, affect the splicing pattern of genes, and regulate gene expression. For example, lncRNA Pnky binds to the splicing factor PTBP1 and regulates the splicing pattern of genes involved in neurogenesis [26].

In this study, we confirmed that CAR10 is upregulated in cervical cancer tissue and promotes proliferation of cervical cancer cells. However, its mechanism is still unclear. Therefore, we explored the mechanism of CAR10 in cervical cancer from the direction of ceRNA mechanism. The mechanism by which miRNA regulates gene expression is to bind to the miRNA reaction element (MRE) of the target gene 3′UTR or the 5′UTR region, resulting in the complementary binding of miRNA to MRE, and directing the RISC to silence or degrade the target mRNA, inhibiting gene transcription [27, 28]. However, in recent years, studies have found that in addition to the traditional miRNA-RNA regulatory mechanisms, there is a reverse RNA-miRNA regulation mechanism, namely, the competitive endogenous RNA (ceRNA) hypothesis. ceRNA can compete for binding to miRNAs, affecting the regulation of miRNAs on target gene mRNA, thereby regulating the expression of target genes. Any RNA containing MRE may be ceRNAs, including pseudogenes, mRNAs, and lncRNAs [29, 30]. Among them, lncRNA, as a ceRNA, plays an important role in the development of cervical cancer by sponging miRNA to regulate expression of genes. For example, lncRNA TUSC8 can upregulate the expression of PTEN by sponging miR-641, thereby inhibiting the invasion and migration of cervical cancer cells [31]. LncRNA MIR205HG can act as a ceRNA to promote the proliferation and migration of cervical cancer cells by reversing the inhibitory effect of miR-122-5p on FOXP2 [32]. LncRNA CDKN2B-AS1 acts as a ceRNA and binds to miR-181a-5p, reversing the inhibitory effect of miR-181a-5p on TGF*β*I, leading to malignant progression of cervical cancer [33]. In ceRNA networks, lncRNA and mRNA contain the same MRE, so lncRNA can compete with miRNA to avoid inhibition of mRNA by miRNA. In this study, we found through bioinformatics prediction that both CAR10 and PDPK1 contain MRE for miR-125b-5p. It was verified by molecular biology experiments that CAR10 can bind to miR-125b-5p. Moreover, both CAR10 and PDPK1 are target genes of miR-125b-5p. Further studies revealed that overexpression of CAR10 reversed the inhibitory effect of miR-125b-5p on PDPK1 expression. These results indicate that CAR10 can act as a ceRNA to promote PDPK1 expression by sponging miR-125b-5p.

Does the CAR10/miR-125b-5p/PDPK1 network participate in the development of cervical cancer? Through in vivo study, we confirmed that CAR10 overexpression can significantly promote the proliferation of cervical cancer cells, suggesting that CAR10 plays a key role in the development of cervical cancer. In addition, in cervical cancer cells, overexpression of miR-125b-5p, or knockdown of PDPK1, can reverse the promotion of CAR10 on cervical cancer cell proliferation. Our study shows that the CAR10/miR-125b-5p/PDPK1 network plays an important role in the development of cervical cancer.

In this study, we found that CAR10 is upregulated in the development of cervical cancer. In cervical cancer cells, CAR10 acts as a ceRNA, which upregulates the expression of PDPK1 by sponging miR-125b-5p, thereby promoting the proliferation of cervical cancer cells. In addition, we also confirmed that intervention of the CAR10/miR-125b-5p/PDPK1 network can inhibit the development of cervical cancer and provide a new strategy for targeted therapy of cervical cancer.

## Figures and Tables

**Table 1:** Clinical characteristics of 40 cervical cancer patients.

Clinicopathologic feature	*n* (%)	CAR10 (mean ± SEM)	*p* value
*Age*			*p*=0.1556
≤50	22 (55.00%)	2.11 ± 0.22	
≥50	18 (45.00%)	2.07 ± 0.24	

*Menopause*			*p*=0.3107
Yes	24 (60.00%)	2.47 ± 0.21	
No	16 (40.00%)	2.38 ± 0.22	

*Histology*			*p*=0.5134
Squamous cell cancer	20 (50.00%)	1.98 ± 0.15	
Adenocarcinoma	14 (35.00%)	2.03 ± 0.19	
Others	6 (15.00%)	2.10 ± 0.2	

*Differentiation*			*p* < 0.0001
Well to moderate	30 (75.00%)	2.14 ± 0.22	
Poor	10 (25.00%)	3.62 ± 0.41	

*Grade number (%)*			*p* < 0.0001
I	6 (15.00%)	1.11 ± 0.18	
II	20 (50.00%)	2.23 ± 0.31	
III	14 (35.00%)	3.51 ± 0.38	

*Tumor size*			*p*=0.0204
<4 cm	26 (65.00%)	2.35 ± 0.27	
≥4 cm	14 (35.00%)	2.16 ± 0.30	

*Lymph node*			*p*=0.0125
Negative	30 (75.00%)	2.12 ± 0.21	
Positive	10 (25.00%)	2.21 ± 0.18	

## Data Availability

The data used to support the findings of this study are included within the article.

## References

[1] Lei J., Andrae B., Ploner A. (2019). Cervical screening and risk of adenosquamous and rare histological types of invasive cervical carcinoma: population based nested case-control study. *BMJ*.

[2] Santamaria-Ulloa C., Valverde-Manzanares C. (2019). Inequality in the incidence of cervical cancer: Costa Rica 1980-2010. *Frontiers in Oncology*.

[3] Luciani S., Bruni L., Agurto I., Ruiz-Matus C. (2018). HPV vaccine implementation and monitoring in Latin America. *Salud Pública de México*.

[4] Kondo Y., Shinjo K., Katsushima K. (2017). Long non-coding RNAs as an epigenetic regulator in human cancers. *Cancer Science*.

[5] Li M., Duan L., Li Y., Liu B. (2019). Long noncoding RNA/circular noncoding RNA-miRNA-mRNA axes in cardiovascular diseases. *Life Sciences*.

[6] Castellanos-Rubio A., Ghosh S. (2019). Disease-associated SNPs in inflammation-related lncRNAs. *Frontiers in Immunology*.

[7] Barangi S., Hayes A. W., Reiter R., Karimi G. (2019). The therapeutic role of long non-coding RNAs in human diseases: a focus on the recent insights into autophagy. *Pharmacological Research*.

[8] Li Y., Jing F., Ding Y., He Q., Zhong Y., Fan C. (2018). Long noncoding RNA CCAT1 polymorphisms are associated with the risk of colorectal cancer. *Cancer Genetics*.

[9] Yu Q., Zhou X., Xia Q. (2017). Long non-coding RNA CCAT1 that can be activated by c-Myc promotes pancreatic cancer cell proliferation and migration. *American Journal of Translational Research*.

[10] Chen X., Zeng K., Xu M. (2018). SP1-induced lncRNA-ZFAS1 contributes to colorectal cancer progression via the miR-150-5p/VEGFA axis. *Cell Death & Disease*.

[11] Xie S., Ge Q., Wang X., Sun X., Kang Y. (2018). Long non-coding RNA ZFAS1 sponges miR-484 to promote cell proliferation and invasion in colorectal cancer. *Cell Cycle*.

[12] Zhang X., Yao J., Shi H., Gao B., Zhang L. (2019). LncRNA TINCR/microRNA-107/CD36 regulates cell proliferation and apoptosis in colorectal cancer via PPAR signaling pathway based on bioinformatics analysis. *Biological Chemistry*.

[13] Sun L., Xue H., Jiang C. (2016). LncRNA DQ786243 contributes to proliferation and metastasis of colorectal cancer both in vitro and in vivo. *Bioscience Reports*.

[14] Sun Y., Ma L. (2019). New insights into long non-coding RNA MALAT1 in cancer and metastasis. *Cancers*.

[15] Ge X., Li G.-y., Jiang L. (2019). Long noncoding RNA CAR10 promotes lung adenocarcinoma metastasis via miR-203/30/SNAI axis. *Oncogene*.

[16] Wei M. M., Zhou Y. C., Wen Z. S. (2016). Long non-coding RNA stabilizes the Y-box-binding protein 1 and regulates the epidermal growth factor receptor to promote lung carcinogenesis. *Oncotarget*.

[17] Li K., Ma Y.-b., Zhang Z. (2018). Upregulated IQUB promotes cell proliferation and migration via activating Akt/GSK3β/β-catenin signaling pathway in breast cancer. *Cancer Medicine*.

[18] Li K., Xu X., He Y. (2018). P21-activated kinase 7 (PAK7) interacts with and activates Wnt/β-catenin signaling pathway in breast cancer. *Journal of Cancer*.

[19] Wang C., Wang L., Ding Y. (2017). LncRNA structural characteristics in epigenetic regulation. *International Journal of Molecular Sciences*.

[20] Pandey R. R., Mondal T., Mohammad F. (2008). Kcnq1ot1 antisense noncoding RNA mediates lineage-specific transcriptional silencing through chromatin-level regulation. *Molecular Cell*.

[21] Ma S., Ming Z., Gong A.-Y. (2017). A long noncoding RNA, lincRNA-Tnfaip3, acts as a coregulator of NF-κB to modulate inflammatory gene transcription in mouse macrophages. *The FASEB Journal*.

[22] Zhang X., Zhao X., Li Y., Zhou Y., Zhang Z. (2019). Long noncoding RNA SOX21-AS1 promotes cervical cancer progression by competitively sponging miR-7/VDAC1. *Journal of Cellular Physiology*.

[23] Sawaya A. P., Pastar I., Stojadinovic O. (2018). Topical mevastatin promotes wound healing by inhibiting the transcription factor c-Myc via the glucocorticoid receptor and the long non-coding RNA Gas5. *Journal of Biological Chemistry*.

[24] Mayama T., Marr A., Kino T. (2016). Differential expression of glucocorticoid receptor noncoding RNA repressor Gas5 in autoimmune and inflammatory diseases. *Hormone and Metabolic Research*.

[25] Liu Y. W., Xia R., Lu K. (2017). LincRNAFEZF1-AS1 represses p21 expression to promote gastric cancer proliferation through LSD1-Mediated H3K4me2 demethylation. *Molecular Cancer*.

[26] Ramos A. D., Andersen R. E., Liu S. J. (2015). The long noncoding RNA Pnky regulates neuronal differentiation of embryonic and postnatal neural stem cells. *Cell Stem Cell*.

[27] Rouleau S., Glouzon J.-P. S., Brumwell A., Bisaillon M., Perreault J.-P. (2017). 3′ UTR G-quadruplexes regulate miRNA binding. *RNA*.

[28] Andreou A. Z., Harms U., Klostermeier D. (2019). Single-stranded regions modulate conformational dynamics and ATPase activity of eIF4A to optimize 5′-UTR unwinding. *Nucleic Acids Research*.

[29] Grüll M. P., Massé E. (2019). Mimicry, deception and competition: the life of competing endogenous RNAs. *Wiley Interdisciplinary Reviews: RNA*.

[30] Hu X., Yang L., Mo Y. Y. (2018). Role of pseudogenes in tumorigenesis. *Cancers*.

[31] Zhu Y., Liu B., Zhang P., Zhang J., Wang L. (2019). LncRNA TUSC8 inhibits the invasion and migration of cervical cancer cells via miR-641/PTEN axis. *Cell Biology International*.

[32] Li Y., Wang H., Huang H. (2019). Long non-coding RNA MIR205HG function as a ceRNA to accelerate tumor growth and progression via sponging miR-122-5p in cervical cancer. *Biochemical and Biophysical Research Communications*.

[33] Zhu L., Zhang Q., Li S., Jiang S., Cui J., Dang G. (2019). Interference of the long noncoding RNA CDKN2B-AS1 upregulates miR-181a-5p/TGFβI axis to restrain the metastasis and promote apoptosis and senescence of cervical cancer cells. *Cancer Medicine*.

